# On the Treatment of Fractures, &c., by the Use of the Roller

**Published:** 1850-06

**Authors:** 


### REVIEWS.

Art. V.—*On the Treatment of Fractures, &c., by the use of the Roller.* By B. W. Dudley, M. D., Professor of Surgery in Transylvania University. Transylvania Medical Journal, 1850. Vol. 1, No. v.

The paper before us embraces a subject which has occupied a considerable portion of this Journal within a few months past; we shall not therefore, enter upon an elaborate analysis of the paper, nor weary our readers with the many reflections that naturally arise from the perusal of an essay, combining some merit, with numerous faults; thrown together with seeming disregard to system or order, and marred on every page by errors, that reflect most seriously either on the author or proof reader, perhaps on both. The practical import of the essay may be expressed in a single word—the roller; whether the fracture be of an arm or leg—of the neck of the femur or scapula, each one requires and receives but a single remedy.

Of all the accidents to which the bones are liable, fractures are, by far, the most frequent; none demand more prompt and careful attention, nor illustrate more clearly the importance and utility of the surgeon's art. To the surgeon nothing can be of more moment than a clear understanding of the nature of these accidents, without which he must often inevitably fail in discharging with skill, the duty that fractures require of him. A mistake may be fatal to his reputation; for a distorted limb will be considered as a living monument of his want of skill, and is but too often an enduring, and should ever be, a mortifying reproach to the surgeon.

It is but reasonable to suppose that these accidents were among the very first that awakened the solicitude, and gave exercise to the ingenuity of the great progenitors of our race. Certain it is, that in the most ancient records of medical science, we find very minute and complicated directions for the management of these serious casualties; mechanical genius has in all ages been called into requisition, and the inventive power of man has, in no department of surgery, shown itself so fruitful as in this. Screws, clamps, splints of every imaginable shape and material, bandages innumerable, and every contrivance, that was supposed to be capable of repairing the injury, or of adding to the quiet and comfort of the sufferer, meet us in surgical records.

But the bed of Hyppocrates, and the Glosocomon, as used by the ancients, and pictured in engravings in the works of the great master Paré, have been abandoned on account of their complexity. Avicenna's method has shared the same fate, although it was approved, and in some degree modified, by Petit, Heister, and Duverney. The machines of Bellocq, Hook, and countless others have deservedly fallen into disuse. Desault's plan, more simple and practicable, was yet beset by so many difficulties that it could not be sustained, even when supported by the improvements of the distinguished Boyer and others. These innumerable appliances, that have rapidly succeeded each other in every age, have probably owed their existence to the infinite variety of the accidents, which they were intended to remedy. There are striking differences in fractures as they occur in different bones, or in different parts of the same bone; in regard to the direction of the fracture; the relative position of the fragments; and in the attending circumstances, which may present the fracture to us as simple or compound.

But varied as these conditions may be, the indications are quite simple. If displacements accompany the accident, the indications are: 1st. To readjust the separated fragments. 2d. To maintain them in proper position until they are united by ossific matter, and, 3d. To anticipate such accidents as are likely to happen, or to meet them with suitable remedies when they have already supervened.

The more simple the means by which these purposes are effected, the better, and the tests to which we would submit any mechanical contrivance, for securing fractured limbs, are their simplicity—promise of comfort, and success. Thus tested, we believe the bandage, as commended at various times by some of the ablest men of the profession, is infinitely superior to all the expensive, cumbrous, yet ingenious machinery, that in former times, stood in fearful array in almost every surgeon's office; and we cannot but be surprised, that while so many able and intelligent writers seem to have appreciated the importance of this simple but powerful agent, few availed themselves of all the benefits that flow from its judicious application.

But the numerous mechanical contrivances, to which we have alluded, should not be discarded without consideration; students should ever bear in mind the judicious advice of Charles Bell "not to pin their faith upon any individual master, school, or hospital, as is too often the case; but look around them, and particularly learn what our predecessors have said and done." Half a century since, one of England's most distinguished surgeons and physiologists, unblushingly declared, that "reading was folly," and but too many of his ardent admirer's discovered, when too late, that the greater part of their lives had been thrown away, in the futile effort, to wring from nature, truths long since brought to light by those very men whose labors they were taught to scorn and deride.

The spirit of the present age is happily with Mr. Bell, and some study of the literature of the profession has become an imperious necessity; while nature is appealed to as a test of truth, and consulted with a view to further accessions to the common stock. Hence we are no longer in danger of losing what is old and good, no matter how much we gain in our search after novelty. "Those who are not aware of the observations and discoveries of the great men who have preceded them, are in continual danger, in straining after new inventions, of only restoring what has been discovered, tried, and rejected before their time." Hence the importance of looking into, even, the errors of the past, that we may wisely steer clear of the shoals that may have proved disastrous to others. In no department of medicine is this of so much moment, as in that which regards the treatment of fractures. How often has the same instrument been ignorantly reproduced, and at the cost of how much of human suffering? Truth and error should be alike closely analysed, for, thoroughly comprehended, both serve to direct our steps in the pathway of duty. Having examined a great number of instruments for dressing fractures, and witnessed the application of not a few, we take pleasure in awarding to our author the honor of contributing efficient aid to the revival and extension of the use of the bandage as a substitute for them all; for we repeat, that in our opinion, with the addition of simple splints, the roller is, beyond all question, the most simple and perfect means in the hands of the profession. It is alike convenient to the surgeon and comfortable to the patient. We are free to confess, however, that we have not found it practicable to dispense with splints; nor do we know any one, except our author, who would feel authorised to rely wholly on the bandage. Professor D. declares that he has not used a splint, save in fractures of the forearm, "for thirty years past in practice," and then only for the purpose of preserving the space between the radius and ulna, so absolutely necessary to the motions peculiar to that part of the body. We presume there is some mistake here, since the Professor says, on page 446 that: "in consequence of the obliquity of the fracture, splints eight inches long were directed to be tied on" in a case that occurred "within the past year." This is certainly an admission that splints do sometimes become necessary, and in this opinion there is a unanimous concurrence amongst all surgeons with whose writings or opinions we are acquainted. Although "the bandage," to use the language of Boyer, "instead of occasioning an edematous swelling of the limb, obviates it by making equable pressure on all its parts, which acts in direct opposition to the muscles, that tend to separate the portions of bone, and which, by acting on these muscles, diminishes their irritability and enfeebles their action, must concur powerfully with a good position to effect union of the divided portions;" yet in the varied motions of the patient, the mere weight of the limb, unsupported by something less yielding than the roller, would be liable to produce curvature, that must ever after remain an irremediable injury to the patient, and an enduring reproach to the surgeon." In view of the very clear notions of the effects and advantages of the roller, in certain fractures, entertained by Boyer, and before his time proclaimed by others, we are not a little surprised, that he failed to apply, the same sound and wholesome principles, to the management of the same accidents in every part of the body, unincumbered by the complicated, inconvenient and painful machinery to which he too often resorted. Take, for example, his treatment of fractured tibia; and we will venture to assert that neither any of his predecessors or successors, have ever devised a plan, more simple in character, or better calculated to promote the comfort of the patient, and to secure the restoration of the limb to symetry and soundness. "A long compress placed on the anterior part of the leg, and over that a roller or circular bandage, with which the leg being covered, three splints of pasteboard, or thin wood, are applied and bound on by the part of the roller which remained unapplied." The value of this mode of dressing fractures, and its vast superiority over all others, was most ably presented and illustrated by cases, in two essays, by one of the editors of this Journal in the February and April numbers of the present year, to which, we take great pleasure in refering our readers, as the best articles, on the subject of the bandage, that we have had the good fortune to peruse. If our readers will do us the favor to turn again to these essays, they will perceive, that it would be a work of supererogation on our part to extend our remarks on the essay before us; we shall therefore content ourself with a brief analysis of our author's views, accompanied by a very few remarks of our own.

The first paragraphs shadow forth the practical teachings of the whole essay, and as our readers must have already anticipated, these are simple, easily understood, and in strong contrast with the complicated and tormenting machinery too common in former times, to which we have alluded, and which has happily long since gone down to oblivion, or is now only occasionally recalled as warning to ourselves, and as fruitful evidence of the ingenuity of able men, whose whole lives were devoted to ardent and unwearied efforts to alleviate human suffering.

"The benefits arising out of the skillful application of the roller to the treatment of fractures, have at no period of surgical history united the confidence and commendation of enlightened surgeons in a measure commensurate with its merits." So many mischievous errors have been propagated in regard to this fatal agent, "that surgeons have been more watchful to guard against apprehended injuries consequent to its application, than prompt in embracing the advantages of a remedy, which must be classed amongst the most powerful, the least equivocal, and the most rational in the profession."

The objections of our author to fracture machinery, generally, are well laid, and their force will be fully appreciated by all who have attempted to avail themselves of their promised benefits.

The "position of the fractured limb" is a matter of considerable import; and while most surgeons prefer the semiflexed position, experience has demonstrated its inconvenience and impolicy in many cases—as, for example, in fractured patella. Having determined to resort to the roller:

"The time and manner of its application are subjects especially worthy of consideration, and of the exercise of skill. All the muscles, which are to be placed under its control, should be thoroughly extended, provided they are in a state of preternatural contraction, preparatory to the application of the roller; while the force exercised in applying it, must be in proportion to the amount of cellular tissue in the limb, or of the interstitial deposite, which may have been the product of effusion, or of inflammatory action in the part."

An avoidance of "pain and suffering," whether the patient "is resting quietly upon a couch, or subjected to the flying ambulance upon field of battle;" the ease and facility of removing the dressings; the support given to the soft parts, often shocked "beyond their capabilities for healthy reaction;" the control exerted over "destructive inflammatory excitement;" the power to retard mortification and sloughing when present, are offered as so many reasons in support of the Professor's favorite remedy.

Sir Astley Cooper's circular bands, and Mr. Lonsdale's iron clamps in fractured patella, the Professor thinks "render deformity, and imperfect use of the part almost certain;" and beside, he says, eight or ten days must elapse before the swelling and inflammation are sufficiently reduced, according to these gentlemen, to justify a resort to the means they recommend. Before this can be accomplished such changes may have occurred as utterly to preclude all hope of a perfect reunion.

John Bell was the first to publish the opinion that bony reunion does not take place within a capsular ligament; the same doctrine was taught and published by Sir A. Cooper; but he is not so exclusive, in this matter, as is indicated by our author; nor does he inculcate the necessity of delay, in the application of the bandage, in all cases.

The principle taught by Sir Astley was that "ligamentous union extends to all detached portions of bone within a capsular ligament; its vitality being supported merely by the ligament within the joint," but he distinctly admits the possibility of bony reunion of fractured patella. His attention was attracted to this subject by a discovery made in dissecting a body at St. Thomas's hospital; both patella had been fractured longitudinally, and although the fragments of each were almost in contact, ossific union had taken place in neither. This surprised Sir Astley, and he immediately entered upon a series of experiments, on animals, for the purpose of elucidating this phenomenon. These experiments resulted in proving that the failure of ossific reunion, after fracture of this bone, was owing to more or less separation of the fragments, and that, when they were kept in actual contact, ossific agglutination took place. In compound fracture of this bone, this distinguished surgeon inculcated the wholesome principle that the prime "object is to produce adhesion immediately, and every means in our power must be used to effect it." He applied the bandage without delay, and thus succeeded, with the aid of a few stiches and adhesive strips, in effecting cures in several cases. In recent simple fracture, where no swelling, etc., had supervened, there is no material difference between the treatment proposed by Sir Astley, and that pursued by our author; where there was much swelling, the former gentleman did not appreciate the bandage so highly as it deserves, but thought proper to resort to depletion, and evaporating lotions for a few days before a resort was had to the roller. In our opinion, our author is correct in considering the bandage highly useful at every period of the treatment, and when there is much swelling, pain, etc., it is more imperiously demanded than under any other circumstances.

The following is our author's treatment of fractured patella:

"The limb was sponged with warm water, and two rollers were made wet with spirit. A narrow roller was evenly applied, without force, from the toes to the knees, and then made fast. When the superior fragment of the bone had been brought into place by an assistant, with the aid of pressure applied upon the front and upper part of the thigh, a broad roller, after being fixed around the hips, was brought down upon the thigh. With every successive turn of this, the soft parts were pressed gently before it, and finally presented a superfluity about the knee, when this roller was made fast. It now remained to adjust the fragments in apposition, and to give them proper protection. With this view the roller upon the leg was continued over the knee. By oblique turns of this, made to embrace the superior and inferior fragments alternately, they were approximated, and when fairly adjusted, the obliquity of the roller was regularly diminished, until by transverse turns, equable and efficient compression was applied to all points of the surface."

The dressings remained undisturbed until the tenth day, when interstitial absorption had progressed so far as to render their removal and reapplication necessary. "At the end of the third week, ossification between the fragments being complete, the dressings were removed the second and last time." Two weeks afterwards the same bone was refractured, and was subjected to a similar treatment;" at the end of the sixth week ossific union was complete, attended by a profusion of ossific deposits; which disappeared some months after, under the use of the limb."

An exuberent ossific secretion is not an unfrequent attendant on fracture, and "such an accident may be converted into an occasion of confering increased length upon the broken bone."

This is illustrated by reference to cases of "*the bone fellon*," in which, after the terminal phalanx was "wrenched from its connexions with the capsule, ligaments, and cartilage of the joint," a new and perfect bone, of increased length, was regenerated, under the influence of the bandage.

The roller is believed to present "the greatest security against artificial joint," and, together with friction, the most efficacious remedy for that important accident.

For the rupture of the tendo-Achillis, or of the tendon uniting the patella to the tibia, the same treatment is regarded as altogether sufficient.

Much discussion has arisen in regard to the possibility of perfect reunion after fractures of the cervix femoris; many of the ablest men of the profession have thought recovery without shortening of the limb and lameness impossible; amongst these are Hildanus, Plantner, Ludwig, Louis, Sabatier, Cooper, Bell, etc. Our author correctly sides with Desault, Boyer, and others, who stoutly maintain that a cure without lameness or deformity may be obtained, and also, a perfect consolidation. Dupuytren says that bony reunion in extra-capsular fracture, may and does often take place; as conclusive proof of the truth of this position he confidently appeals to many specimens preserved in the museum of the Faculty of Medicine, and at the Hospital de la Pitié.

Two of the most common causes of non-consolidation, when the fracture is skillfully dressed, are old age and a complete rupture of the orbicular ligament. Old age is always a serious impediment to the restorative process of nature; and if the orbicular ligament be ruptured, the head of the bone is effectually cut off from all supplies, and of course must die. The operation of these causes, too often overlooked, has probably been principally instrumental in establishing the mischievous error which regards deformity and lameness as the necessary consequences of this accident. Although old age is generally a serious obstacle, yet it should be borne in mind, that it is not always an insuperable bar to perfect recovery; we might cite many cases to establish the requirement of duty—that an effort should always be made to effect consolidation, however advanced in life the patient may be. Boyer happily succeeded with a patient, who was an octogenarian, and our author had a like success, twice with the same patient, who had reached the age of seventy. The following is the treatment pursued by our author.

"A roller of appropriate width, made wet with spirit, was applied smoothly and evenly to the foot and leg, and made fast upon the knee. Another wider roller, prepared in a similar manner, was made fast around the hips, in anticipation of extension and counter-extension."

"When the muscles had fairly yielded to the powers of extension, as was indicated by the restoration of the length of the limb and facility of abduction and adduction of the toes, the bandage upon the hips was brought down over the superior and back portion of the glutei, around the superior part of the thigh, and again over the hips. In each successive turn around the hips, nates and thigh, care was taken to embrace every portion of the glutei, and deeper seated abductors, together with the opposing muscles on the inner and front portion of the limb. When the pressure here was made equable and uniform in all its bearings, the roller was continued down, and united to that already placed upon the foot, leg, and knee. Although pressure could not be directed immediately upon the psoas, and obturator, the iliacus and pyriformis muscles, involved in this injury; yet the object was accomplished, as in other cases, and by other surgeons where extension and counter-extension is exclusively relied upon; by acting upon the points of their insertion with the roller. The patient was then placed upon his couch, and the rollers were permitted to remain undisturbed for a week, when it became necessary, from the reduced size of the limb, and the loose state of the bandages, to remove and reapply them as before. During a confinement of six weeks, the rollers were removed and reapplied five times, after which he regained the use of his limb, under the effects of gentle exercise upon a crutch."

It is worthy of remark that this cure was effected in less than one half the time usually thought to be necessary to consolidate a fracture of the cervix femoris.

Our author admits the concurrence of all surgeons in the use of the bandage in fractures of the ribs and clavicle; but remarks "that these two species of fracture do not, either of them, exemplify the princinciple upon which its actions depend." The treatment of fractured clavicle is exceedingly simple:

"A sling made of a Bandanna handkerchief; or what may answer a better purpose, a piece of muslin, from three to four feet long and from twelve to fifteen inches wide, with a slit at either end, is proposed with a view to supersede all other dressings."

The following passage imparts a caution which should not be overlooked:

"But the potency of the roller is liable to be abused. Its mischievous effects when applied to inflammatory swellings proceeding from constitutional causes, whether these swellings and inflammations have been invited to the particular location by violence or otherwise, *is* familiarly known; and in such an event the inflammatory action is liable to assume a form not less destructive, and perhaps more painful than the original; even provided a mischievous transfer to parts of more vital importance does not ensue."

Great care is to be exercised, especially in fractures, "attended by extensive laceration of the soft parts, to select a material for the roller, which admits of the most easy access through its texture to the cutaneous surface * * * * the *open coarse cotton*, with a strong thread; surpasses as the material for the roller, all dressings, hitherto suggested in these cases."

The application of a glutinous substance upon the surface of the roller, as has been suggested by some of the French surgeons, is reprobated in strong terms as being well calculated "to divest the remedy of all its virtues."

The following course was successfully resorted to in a case of fistulous abscesses, and an artificial joint about the middle of the humerus:

"The parts were handled with great freedom in order to expel all purulent secretions from the abscesses, and at the same time to cause free and forcible friction between the broken ends of the humerus. So soon as these objects were accomplished, a roller was put on from the fingers to the elbow, and then in a flexed position of this joint, the roller was continued over the elbow and as high as the shoulder. The forearm being placed in a sling, the patient was directed to return the next day."

This dressing was renewed daily for a week, until "the fragments of the bone had become so sensitive as to forbid further disturbance." At the end of the fourth week ossific union was complete.

The following is an interesting, and certainly an extraordinary case:

"Mr. B. was brought on a mattrass to this place, and examined twenty-four hours after having his hand, fore-arm, and arm lacerated and crushed, between a heavy pair of iron cylinders while they were in rapid motion. Several severe lacerations were inflicted on the hand. A lacerated incision extended from a little above, and upon the outside of the wrist, nearly up to the elbow, involving and injuring extensively the interosseous ligament. The radius and ulna were thoroughly ground into small fragments, and these were intermingled.

"By means of the fingers and thumbs of both my hands, all of which were admitted together down to the interosseous ligament; the fragments of *the* one bone of the fore-arm were separated from those of the other; which could not have been accomplished, except through the facilities afforded by the external wound.

"A profound and irreparable injury, consisting in the death of the whole of the integument of the front surface of the fore-arm, was then exposed to view; and as the sequel proved, this was attended by a like condition of the superficial common flexor of the fingers. The humerus was extensively crushed, yet not so much ground up as were the bones of the fore-arm; nor was it a compound fracture. The axilla, the breast, the side, and the shoulder, by checking the impetus of the machinery, suffered great and alarming contusions and exhibited extensive effusion of blood."

"When the injured surfaces had been for half an hour under the influence of hot spirits, as an ablution, the dressings were made ready. These consisted of a roller and two pledgets made wet with spirits, with two narrow splints for the fore-arm, and a roller for the arm."

"Free and frequent ablutions to the whole limb with spirits, were ordered, for the purpose of washing off bloody effusions from the surface of the dressings; which were not removed until the twelfth day."

At the end of four months "the wound was cicatrised and the dressings discontinued;" the use of the arm and hand was almost perfectly restored.

Fractures of the fore-arm should always be dressed with the hand in supination, and to secure this position, care should be taken to apply the roller from the ulnar side over the front of the arm. Suitable compresses, to preserve the proper space between the two bones, should never be neglected.

The essay under review, contains several very interesting and instructive cases, which we should be pleased to present to our readers, but they are scarcely susceptible of condensation, and our space will not permit us to give them in full. They all, however, teach one doctrine, that no case can be so desperate—no laceration so extensive and terrible, as to preclude the hope of preserving the limb under the influence of the bandage.

We do not feel an implicit faith in the roller alone in all cases, but we are convinced that in most fractures, with the aid of a few simple splints of stiff pasteboard or thin wood, it is infinitely superior to all the fracture machinery ever invented, being alike simple in its application, always accessible, safe and comfortable to the patient, and serving as an effectual bar to many of the most distressing and troublesome accidents that so frequently complicate these injuries.

Our author is not original in the application of the roller in the treatment of fractures, but he stands almost or quite alone in discarding all other means. While a vast majority of surgeons have resorted to more or less machinery in dressing fractures, many of the ablest writers, from Galen to the present day, have borne testimony to the potency of the bandage, and simplicity in the management of these accidents has now become almost universal. We have not deemed a reference to these authorities necessary, on the present occasion, inasmuch as they have already spoken in the two essays of Dr. Bell, to which we have invited the attention of our readers. We may be indulged, however, in quoting a paragraph from Dr. Bell, while we refer to his essays for the numerous citations by which he has abundantly fortified his position:

"From the days of Hippocrates and Galen, down to the present, through all the varying schools that have lived and perished, through all the wild, fanciful and chimerical systems and theories that have been in vogue, as well as among the most truthful and observant of sects, the bandage has steadily maintained its position, as, at once, not only the most useful and valuable of all agents for its purposes, but the one of all others, capable of the most general and extensive use. The highest authorities in all ages of medicine, have constantly endeavored to bear testimony to its great qualities, and many of the old surgeons had as clear and extended views of its multiform capacity for a great variety of surgical purposes, as any of the most enlightened among the moderns. Why should there, then, be any hesitation about the use of a remedy thus commended? If the accumulated experience of many centuries is of any service, why seek to deviate from that plain faith that is studded with monuments of success, and that has all the leading lights of the medical profession playing upon it?" C.

